# Progression of kidney disease in type 2 diabetes – beyond blood pressure control: an observational study

**DOI:** 10.1186/1471-2369-6-8

**Published:** 2005-06-28

**Authors:** David J Leehey, Holly J Kramer, Tarek M Daoud, Maninder P Chatha, Majd A Isreb

**Affiliations:** 1 Departments of Medicine Veterans Affairs Hospital Hines, IL and Loyola University Medical Center Maywood, IL, USA; 2 Department of Preventive Medicine, Loyola University Medical CenterMaywood, IL, USA

## Abstract

**Background:**

The risk factors for progression of chronic kidney disease (CKD) in type 2 diabetes mellitus (DM) have not been fully elucidated. Although uncontrolled blood pressure (BP) is known to be deleterious, other factors may become more important once BP is treated.

**Methods:**

All patients seen in the outpatient clinics of our hospital between January 1993 and September 2002 with type 2 DM and clinical evidence of CKD were evaluated. Progression of kidney disease was evaluated by rate of decline of glomerular filtration rate (GFR) as estimated from the simplified MDRD formula. Variables associated with progression in univariate analyses were examined by multivariate analysis to determine the factors independently associated with kidney disease progression.

**Results:**

343 patients (mean age 69 years; all male; 77% Caucasian) were studied. Mean BP, glycated hemoglobin, and serum cholesterol during the study period were 138/72 mmHg, 8.1%, and 4.8 mmol/L, respectively. Mean decline of GFR was 4.5 ml min-1 1.73 m^2^-1 yr-1 (range -14 to +32). Low initial serum albumin (p < 0.001), black race (p < 0.001), and degree of proteinuria (p = 0.002), but not blood pressure, glycated hemoglobin, or serum cholesterol, were independently associated with progression.

**Conclusion:**

In a cohort of diabetic patients with CKD in whom mean BP was < 140/80 mmHg, the potentially remediable factors hypoalbuminemia and proteinuria but not blood pressure were independently associated with progression of kidney disease. Further understanding of the relationship between these factors and kidney disease progression may lead to beneficial therapies in such patients.

## Background

Diabetic nephropathy is the most common cause of end-stage kidney disease (ESKD) in the United States and worldwide. Most diabetic patients with ESKD have type 2 diabetes. Since only a minority of type 2 diabetic patients develop kidney disease, predisposing factors for development of the disease are operative. In addition, once clinical kidney disease is evident, the rate of decline of glomerular filtration rate (GFR) is highly variable, ranging from 2 to 20 ml min-1 yr-1 [1]. The reasons for these differences in the rate of disease progression are multifactorial, including both non-modifiable and modifiable factors [2,3]. Blood pressure control is known to be important in preventing adverse cardiovascular and renal outcomes in diabetic patients with hypertension [4]. However, it is not clear whether or not blood pressure is an important predictor of GFR decline in diabetic patients with CKD in whom blood pressure is controlled.

The purpose of the present study was to determine the factors independently associated with chronic kidney disease (CKD) progression assessed by rate of decline of GFR in a cohort of male predominantly elderly veteran patients with type 2 diabetes. In this population, in whom BP was generally well controlled, hypoalbuminemia, black race, and degree of proteinuria, but not blood pressure, were associated with disease progression.

## Methods

### Subjects

A search of the computerized patient record system at Hines VA Medical Center, Hines, Illinois was performed to find all patients seen in the outpatient clinics between January 1993 and September 2002 with type 2 DM and clinical evidence of CKD. Potential subjects were included if they had all of the following: a) type 2 DM [clinical diagnosis and/or glycated hemoglobin > 6.5%]; b) chronic kidney insufficiency, defined by a persistent elevation of serum creatinine (SCr) level; at least 3 serum creatinine values of > 124 μmol/L with at least a six-month interval between the first and last serum creatinine value were required for inclusion; c) proteinuria [positive urine dipstick or urine protein > 0.15 g/d or urine albumin/creatinine ratio ≥ 30 mg/g]. GFR was calculated using the simplified MDRD formula [GFR (ml min-1 1.73 m^2^-1) = 186 × (SCr)^-1.154 ^× (age)^-0.203 ^× (0.742 if female) × (1.210 if black)][5]. The serum creatinine concentration was not calibrated to MDRD laboratory creatinine values. Patients known to have another cause of kidney disease (e.g., polycystic kidney disease, ischemic nephropathy, or biopsy-proven non-diabetic kidney disease) were excluded. In order to determine rate of CKD progression, individual GFR values were calculated from every serum creatinine value between the time when the serum creatinine was first noted to be > 124 μmol/L (baseline value) and either the time of the last available serum creatinine or the commencement of kidney replacement therapy. The rate of GFR decline (slope) (ml min-1 1.73 m^2^-1 yr-1) for each patient during the study period was then determined by linear regression analysis using all calculated GFR values. Decline in GFR was represented as a positive value. The mean duration of the study period was 36 months (range 7–149). The mean number of GFR values utilized for slope determination was 14 (range 3–69).

### Co-variates

Information on age, race, weight, height, use of angiotensin converting enzyme (ACE) inhibitors or angiotensin receptor blockers (ARBs), blood pressure, glycated hemoglobin, serum cholesterol, hemoglobin, serum albumin, and urine protein excretion was obtained from the computerized patient record system. Age, weight (kg) and height (m) were defined as the recorded values at the time of the first laboratory creatinine measurement > 124 μmol/L. Body mass index (BMI) (kg/m^2^) was then calculated using the weight and height values. Race was self-reported. Blood pressure was measured at each clinic visit by a medical technician or a nurse (generally every 3–6 months) in the seated position using a mercury sphygmomanometer. Glycated hemoglobin was measured by high performance liquid chromatography. Hemoglobin values were measured by Coulter counter. Serum values of creatinine, cholesterol, and albumin were measured by automated analyzer. Urinary protein was measured by the pyrogallol red-molybdate method and urinary albumin by immunoturbidemetry. The degree of proteinuria was classified as mild, moderate, or heavy. Mild proteinuria was defined as dipstick trace-0.3 g/L and/or protein 0.15–0.5 g/d and/or spot urine albumin/creatinine ratio of 30–300 mg/g; moderate proteinuria was defined as dipstick 1.0 g/L and/or protein 0.5–3.0 g/d and/or spot urine albumin/creatinine ratio of 300–2000 mg/g; and heavy proteinuria was defined as dipstick 3.0 g/L and/or protein > 3.0 g/d and/or spot urine albumin/creatinine ratio of > 2000 mg/g.

### Statistical analysis

Mean values of systolic blood pressure, diastolic blood pressure, glycated hemoglobin, and serum cholesterol during the study period were computed for each patient and utilized for analysis. Because degree of proteinuria was a categorical variable, maximum degree of proteinuria recorded during the study period was utilized. Since hemoglobin and serum albumin values were expected to decline as kidney disease progressed (see Discussion), initial hemoglobin and serum albumin values (i.e., values obtained closest in time to the beginning of the study period for each patient) were used. Univariate analyses were performed using t-test for continuous variables and Chi-square analysis for categorical variables. Multivariate linear regression analyses using rate of decline (slope) of GFR as the dependent variable were then performed in both age-adjusted and non-age-adjusted fashion using an automated backwards elimination procedure to delete factors with a P-value > 0.15 from the model one at a time. Multivariate analyses were performed using all variables as well as using only those variables found to be significantly associated with progression on univariate analysis.

Data are expressed as mean ± SD. All P values were two sided and a P value of <0.05 was used to indicate statistical significance. Statistical analyses were performed using Systat software (SPSS, Chicago, IL).

This study complied with the recommendations of the Declaration of Helsinki and was approved by the local Institutional Review Board.

## Results

Demographic and clinical variables in the 343 patients who met study criteria are given in Table 1. The mean study period was 36 months (range 7–149). The mean age was 69 (range 43–92) with 73% of patients aged 65 or older. The majority of patients (77%) were Caucasian. Mean values for blood pressure, glycated hemoglobin, and serum cholesterol during the study period were 138/72 mmHg, 8.1%, and 4.8 mmol/L, respectively. The majority of patients (71%) were treated with ACE inhibitors or ARBs.

**Figure 1:**
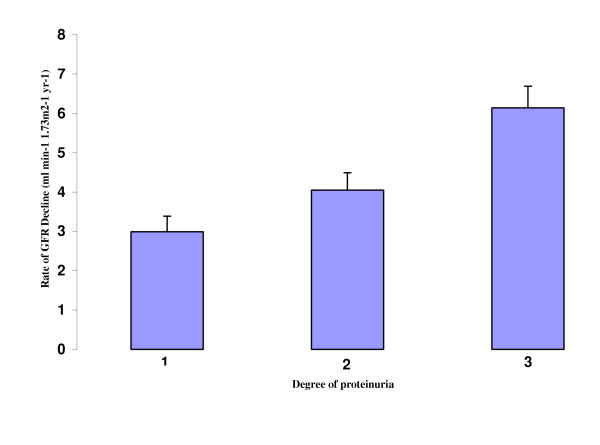
Relationship between degree of proteinuria and rate of GFR decline (1 = mild proteinuria; 2 = moderate proteinuria; 3= heavy proteinuria; see text for definitions)

**Figure 2:**
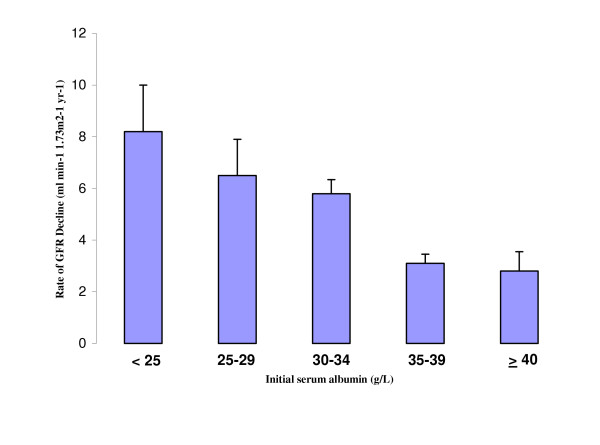
Relationship between initial serum albumin level and rate of GFR decline.

**Figure 3:**
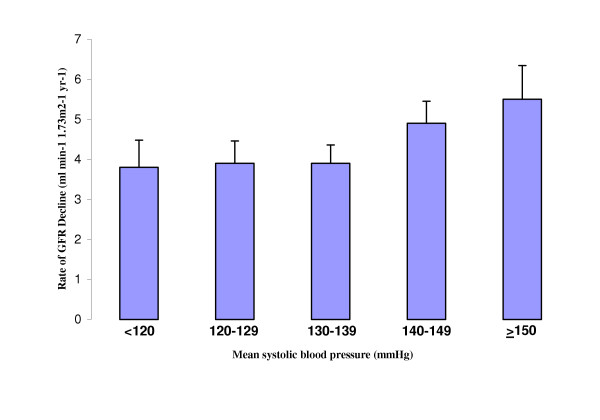
Relationship between mean systolic blood pressure during the study period and rate of GFR decline.

**Figure 4:**
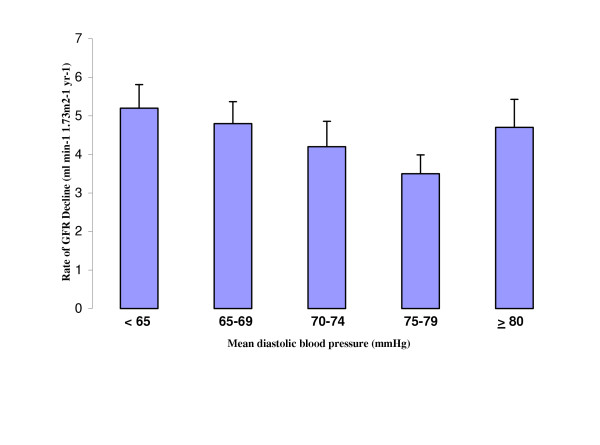
Relationship between mean diastolic blood pressure during the study period and rate of GFR decline.

Mean rate of decline of GFR during the study period was 4.5 ml min-1 1.73 m^2^-1 yr-1 (range -14 to +32). The variables most highly associated with progression on univariate analysis were lower initial serum albumin and greater degree of proteinuria (p < 0.001), followed by younger age and black race (p = 0.002), higher glycated hemoglobin (p = 0.007), higher mean diastolic blood pressure (p = 0.012), lower initial hemoglobin (p = 0.014), and higher systolic blood pressure (p = 0.048) (Table 2). Figures 1 and 2 depict the association between decline in GFR and degree of proteinuria and serum albumin levels, respectively. Patients with greater degrees of proteinuria not unexpectedly had higher rates of disease progression. In addition, the most rapid disease progression was seen in patients with the most profound hypoalbuminemia (serum albumin level < 25 g/L). Rate of progression was only approximately 3 ml min-1 1.73 m2-1 yr-1 with no relationship between serum albumin and progression once serum albumin levels were > 35 g/L. 138 patients (40%) had systolic hypertension (BP = 140 mmHg systolic). There was no association between systolic blood pressure and GFR decline when systolic BP was < 140 mmHg. However, above that level, rate of GFR decline increased with increasing systolic BP (Figure 3). Only 49 patients (14%) had diastolic BP readings of = 80 mmHg. The relationship between diastolic BP and GFR decline was U-shaped, with the lowest rate of progression in the 75–79 mmHg range (Figure 4).

The following independent variables were considered for selection in the stepwise linear regression, using backwards selection: age, race, systolic BP, diastolic BP, glycated hemoglobin, degree of proteinuria, initial hemoglobin, and initial serum albumin level. Because of the U-shaped relationship between diastolic BP and GFR decline, (diastolic BP)^2 ^was also evaluated. When both diastolic BP and (diastolic BP)^2 ^were entered into the regression equation, neither was significantly associated with BP decline (p = 0.943 for diastolic BP and p = 0.935 for (diastolic BP)^2^). In addition, systolic blood pressure and glycated hemoglobin were not independently associated with progression in this analysis. Lower initial serum albumin, black race, and degree of proteinuria were the only variables independently associated with progression at the p < 0.05 level (Table 3). Similar results were seen using a non-age-adjusted model (Table 3) and when all variables regardless of their association with progression were entered into the regression equations.

As regression diagnostics for data from two of the patients indicated high leverage and one was an outlier (with a Studentized residual of 5.071) in the linear regression model, further analyses were performed excluding these cases. The results were not changed after exclusion of these cases, with initial serum albumin (p = 0.001), proteinuria (p = 0.002) and black race (p = 0.003) the only significant variables independently associated with GFR decline.

## Discussion

The most important finding of this study is that the potentially modifiable factors hypoalbuminemia and proteinuria were strongly and independently associated with kidney disease progression in a mostly elderly male type 2 diabetic population with generally well-controlled blood pressure. As shown in Figure 4, there was no effect of systolic BP on progression with systolic BPs < 140 mmHg, and diastolic BP had a U-shaped relationship with GFR decline (Figure 4). Therefore systolic BP and both diastolic BP and (diastolic BP)^2 ^were examined in the multivariate regression analyses. In these analyses, there was no independent association between systolic BP or either diastolic BP or (diastolic BP)^2 ^and rate of GFR decline in this population.

Blood pressure control is known to be important in preventing adverse cardiovascular and renal outcomes in diabetic patients [4]. However, prospective interventional studies demonstrating slowing of kidney disease progression by blood pressure control were mostly performed in type 1 diabetic patients in whom blood pressure was generally poorly controlled by current standards [6-12] (see Table 4). More recent studies in type 1 and type 2 diabetic patients designed to evaluate the effects of blood pressure control on progression have found lesser effects, either because they were underpowered [13] or possibly because of lower pre-treatment and on-treatment blood pressures [14,15]. In support of the latter possibility, in the Reduction of Endpoints in NIDDM with the Angiotensin II Antagonist Losartan (RENAAL) trial, proteinuria, increased serum creatinine, hypoalbuminemia, and anemia at baseline but not baseline blood pressure were predictors of progression [16]. In a recent analysis of RENAAL data, last systolic (but not diastolic) BP prior to end points predicted a higher risk for progressive nephropathy; however, this effect was only seen when systolic BP was > 140 mmHg [17]. In a multivariate analysis of data from the Irbesartan Diabetic Nephropathy Trial (IDNT), another large clinical trial in type 2 diabetic patients, hypoalbuminemia, increased serum creatinine, albuminuria, decreased hemoglobin, and increased blood pressure were predictors in that order of importance [18]. In the ramipril trial of Lewis et al. there was no significant difference in rate of decline in GFR between two groups of type 1 diabetics with mean arterial blood pressures of 98 vs. 92 mmHg, respectively [14]. Similarly, in the ABCD trial, a type 2 diabetes trial, there was no benefit of decreasing diastolic blood pressure from 80–89 mmHg to 75 mmHg (difference in actual BPs 138/86 vs. 132/78, or mean arterial blood pressures of 103 vs. 96 mmHg) [15].

A recent observational study from Japan investigated factors affecting progression of kidney failure in type 2 diabetic patients [2]. These investigators found that lack of insulin therapy, hypoalbuminemia, higher mean blood pressure, and lower hemoglobin at baseline were independent predictors of kidney failure. However, this study involved only 85 patients and a relatively short survey time (14 months), and disease progression was assessed by time to doubling of serum creatinine. Moreover, the mean arterial blood pressure at baseline was 105 mmHg, which is higher than that seen in most recent studies (mean blood pressures during the survey period were not given). In a larger analysis of 227 Caucasian patients with diabetic nephropathy from the Steno Diabetes Center in Denmark, in whom the mean follow-up time was 6.5 years, rate of decline of GFR was predicted by higher values of baseline and mean albuminuria, systolic blood pressure, hemoglobin A1c, and degree of diabetic retinopathy, as well as by baseline GFR, age, lower hemoglobin values, and smoking status during follow-up [3]. In this study, two-thirds of the patients had mean systolic BPs during follow-up of > 149 mmHg. In contrast, in our observational study, the mean BP was 138/72 mmHg (mean arterial blood pressure 94 mmHg) during the study (follow-up) period. This level of BP control was substantially better than that achieved in the Danish study and was also better than that obtained (i.e., 146/76 mmHg) in a recent clinical trial in which a stepped-care algorithm designed to meet American Diabetes Association blood pressure goals of < 130/80 was employed [19]. Thus, taken together, the findings of interventional and observational studies suggest that BP control becomes less important in delaying progression when BP is not very elevated.

The results of our study indicate that hypoalbuminemia independently predicted GFR decline. In a study investigating the relationship between blood pressure and diabetic kidney disease progression, Dillon [20] found that mean serum albumin was associated with decline in GFR. However, since serum albumin could decrease as kidney disease progresses as a consequence of anorexia and malnutrition, this association could be attributed to reverse causality. Using a similar approach to that employed in our study, Ueda et al. [2] reported that baseline serum albumin was a predictor of the development of ESKD in Japanese patients with type 2 diabetes. This study used reciprocal creatinine and/or the development of ESKD as endpoints rather than GFR decline; moreover, the population was quite different from the predominantly Caucasian U.S. male veterans examined in our study. Nevertheless, these data, in conjunction with the RENAAL and IDNT data, in which hypoalbuminemia was also an independent predictor of progression in type 2 diabetes, support the relationship between hypoalbuminemia and GFR decline.

Why would hypoalbuminemia be predictive of kidney disease progression? One possibility is that the serum albumin level reflects the degree of proteinuria, a known risk factor for progression in most kidney diseases, including diabetes [3,21-27]. However, this is not the sole explanation for the association between serum albumin level and disease progression, because hypoalbuminemia is significantly associated with GFR decline after adjustment for the effects of proteinuria. Since many patients with CKD have evidence of chronic inflammation, as reflected by elevated C-reactive protein (CRP) levels, anemia of chronic disease, and elevated D-dimer and vonWillebrand factor levels [28-30], it is possible that hypoalbuminemia may reflect an inflammatory state, which may increase kidney disease progression [30], possibly by causing oxidative stress [31]. In addition, decreased GFR may result in accumulation of proinflammatory compounds (such as advanced glycated end-products and oxidation products) with resultant hypoalbuminemia [32] and kidney injury [33]. The hypothesis that low serum albumin reflects an inflammatory state, which contributes to progression of kidney disease is supported by recent studies in both non-diabetic [34,35] and diabetic [36] populations.

As has been done by other investigators [2,16,18], we utilized initial serum albumin levels in our analyses rather than mean values during the treatment period. Of note, when mean serum albumin values were utilized, there was also a strong association between serum albumin and decline in GFR (data not shown). However, we believed that this could be due to a "reverse" association, as progression of renal failure could result in decreased protein intake, malnutrition, and a decrease in serum albumin level. We also chose to use initial values for hemoglobin rather than mean values during the treatment period. This was done because of the well known direct association between anemia and lower GFR due to erythropoietin deficiency. Mean values of other continuous variables (such as blood pressure) were utilized in order to better capture treatment effects during the observation period and to enhance the precision of the analysis by utilizing many measurements of each variable.

We included patients who had the diagnosis of diabetes mellitus and/or mean glycated hemoglobin values > 6.5% in our analysis. We did not utilize blood glucose values for study inclusion or for analysis since it was not always possible to determine if a sample was a fasting or random sample. We did not find an association between blood glucose control (as reflected by glycated hemoglobin) and progression in our study. The role of hyperglycemia in progression of nephropathy in type 2 diabetes is not clear, in large part because many studies have lacked statistical power. However, in post-hoc analyses of two large clinical trials, RENAAL [16] and IDNT [18], baseline glycated hemoglobin was not an important predictor. However, in their observational study, Rossing et al. [3] recently reported that mean glycated hemoglobin was associated with GFR decline. The lack of association between serum cholesterol and progression in our study is consistent with these other studies [3,16,18].

The observational nature of our study does have limitations. For instance, there was no control over the type of medication used for hypertension, nor any control over diabetes management or dietary intake. We utilized a formula to estimate rate of GFR decline instead of performing direct clearance measurements. The formula utilized, namely the four-variable MDRD estimate, was developed from a largely non-diabetic patient population. Moreover, this formula loses accuracy when the GFR is > 60 ml min-1 1.73 m^2^-1 and/or if the serum creatinine concentration is not calibrated to MDRD laboratory creatinine values [37]. However, our analysis is based on rate of decline of GFR (slope analysis) and not absolute values of GFR and most patients had baseline GFR values of < 60 ml min-1 1.73 m^2^-1. Moreover, the National Kidney Foundation currently recommends using this prediction equation to calculate GFR and monitor progression of kidney disease [5]. Indeed, using rate of GFR decline as our outcome variable has advantages over the doubling of serum creatinine utilized in many prospective clinical trials. This is because doubling of serum creatinine reflects a progressively smaller absolute GFR decline as the serum creatinine increases. Finally, the nature of the kidney disease in our mostly elderly diabetic patients probably represented a mix of diabetic glomerulosclerosis, hypertensive nephrosclerosis, and, in some cases, possibly undiagnosed ischemic nephropathy, either alone or in combination. We did not analyze data on retinopathy since it was not always available in our computerized database. We also did not analyze the relationship between kidney disease progression and cardiovascular disease. Despite these limitations, we believe that our findings are relevant to usual clinical practice.

## Conclusion

The results of our study may have important clinical implications. Although blood pressure control is undeniably important in delaying progression of kidney disease in hypertensive diabetic patients, attention to other potentially remediable factors may be more important in delaying progression once BP is effectively treated. Antiproteinuric agents (such as ACE inhibitors and ARBs) may exert beneficial actions beyond those due to BP control. In addition, the finding that hypoalbuminemia is independently associated with progression of CKD due to type 2 diabetes after adjustment for the effects of proteinuria suggests the possibility that inflammation (and/or malnutrition) may be a progression promoter. In particular, further understanding of the role of inflammation in CKD may lead to therapies which favorably affect kidney disease progression in type 2 diabetic patients.

## Competing interests

The author(s) declare that they have no competing interests.

## Authors' contributions

DJL conceived and designed the study, supervised data collection, carried out the statistical analyses, and prepared the manuscript. HJK assisted in statistical analyses and manuscript preparation. TMD, MPC, and MAI collected data and prepared the spreadsheet for data analysis.

## Pre-publication history

The pre-publication history for this paper can be accessed here:



## Figures and Tables

**Table 1:** Demographic and clinical variables in 343 study patients

Age (years)	69 ± 9 (43–92)
Race	80 black; 263 Caucasian
Body mass index (kg/m2)	30 ± 5 (16–50)
Initial serum creatinine (μmol/L)	150 ± 35 (124–389)
Initial GFR (ml/min/1.73 m^2^)	10 (13–77)
Degree of proteinuria (see text)	Group 1= 118; Group 2 = 100; Group 3 = 125
Mean systolic blood pressure (mmHg)	138 ± 14 (96–189)
Mean diastolic blood pressure (mmHg)	72 ± 8 (49–97)
Treatment with ACEI/ARB	No = 99; Yes = 244
Mean serum cholesterol (mmol/L)	4.8 ± 1.0 (2.3–8.9)
Mean glycated hemoglobin (%)	8.1 ± 1.6 (5.5–17)
Initial hemoglobin (g/L)	128 ± 19 (67–179)
Initial serum albumin (g/L)	34 ± 6 (17–53)

**Table 2:** Univariate association of demographic and clinical variables with decline in GFR

Variable	r	p
Age	-0.192	0.002
Race	*	0.002
Body mass index	-0.010	0.859
Initial serum creatinine	-0.137	0.055
Degree of proteinuria	*	<0.001
Mean systolic blood pressure	0.107	0.048
Mean diastolic blood pressure	0.136	0.012
Treatment with ACEI/ARB	0.039	0.482
Mean serum cholesterol	0.014	0.797
Mean glycated hemoglobin	0.147	0.007
Initial hemoglobin	-0.136	0.014
Initial serum albumin	-0.295	<0.001

*categorical independent variables

**Table 3:** Variables independently associated with rate of GFR decline in multivariate linear regression models

**Age-adjusted:**		
Variable	F ratio	P value

Initial serum albumin	14.5	<0.001
Black race	13.2	<0.001
Degree of proteinuria	10.2	0.002

		
**Non-age-adjusted:**		

Variable	F ratio	P value

Initial serum albumin	15.6	<0.001
Black race	14.4	<0.001
Degree of proteinuria	12.8	<0.001

**Table 4:** Summary of Interventional Studies in Diabetic Patients Examining Effect of Blood Pressure Control on Kidney Disease Progression

AUTHOR	STUDY POPULATION	STUDY SIZE/ DURATION	RESULTS
Mogensen et al. Br Med J 1982 [6]	Type I DM Mean BP 162/103 Urinary albumin 3.9 g/d GFR 86 ml min-1 1.73 m^2^-1	N = 5 73 month treatment period compared to previous 20–31 month untreated period	BP decreased to 144/95 mmHg with treatment Rate of decline of GFR decreased from 14.8 to 5.9 ml min-1 yr-1 in treated period
Parving et al. Lancet 1983 [7]	Type I DM Mean BP 144/97 mmHg Proteinuria > 0.5 g/d GFR approx 77 ml min-1 1.73 m^2^-1	N = 10 39 month treatment period compared to 29 month untreated period	BP decreased to 128/84 mmHg with treatment Rate of decline of GFR decreased from 0.9 to 0.39 ml min-1 mo-1 in treated period
Bjorck et al. Br Med J 1986 [8]	Type I DM Mean BP 163/97 mmHg Mean proteinuria 2.9 g/d Mean serum Cr 2.1	N = 14 4.8 yrs (2.8 yrs untreated followed by 2 yrs treated with captopril)	BP decreased to 155/94 mmHg with treatment (p < 0.02) Urinary protein decreased from 2.9 to 2.8 (NS) Rate of decline of GFR decreased from 10.3 to 5.5 ml min-1 yr-1 (p < 0.02)
Parving et al. Br Med J 1987 [9]	Type I DM > 200 ug/min albuminuria BP 143/96 mmHg pre-treatment Mean GFR approx. 103 ml min-1 1.73 m^2^-1	N = 11 32 mo. untreated followed by 6 yrs treated)	BP decreased to 129/84 mmHg with treatment (p < 0.01) Albuminuria decreased from 1038 to 504 ug/min (p < 0.01) Rate of decline of GFR decreased from 0.89 to 0.22 ml-1 min-1 mo-1 during treatment (p < 0.01)
Parving et al. BMJ 1988 [10]	Type I DM treated with captopril or placebo > 0.3 g/d albumin (mean approx. 0.95) Baseline BP 146/93 mmHg and 137/95 mmHg in 2 groups, respectively Serum Cr < 120 μmol/L GFR mean approx. 97 ml min-1 1.73 m^2^-1	N = 31 Captopril (N = 18) Control (N = 13) 2.5 yrs	MAP decreased by 8.7 mmHg to 136/85 by captopril; increased by 6.6 mmHg to 145/98 mmHg in controls (p < 0.001) Albumin excretion decreased to 390 ug/min in captopril, increased to 1367 ug/min in controls (p < 0.001) GFR decline 5.8 ml min-1 yr-1 in captopril vs. 10 ml min-1 yr-1 in controls (p < 0.01)
Sawicki et al J Diabetes Complications 1995 [11]	Type I DM Proteinuria > 0.5 g/d Serum Cr < 265 μmol/L Diabetic retinopathy BP > 140/90 mmHg or need for BP meds Intensive treatment including BP self-monitoring with goal BP < 140/90 mmHg (IT) vs. routine treatment (RT)	N ~ 100 5 yrs	Primary study endpoints (need for dialysis or death) decreased in IT group
UKPDS 38 1998 13	Type II DM Serum Cr < 177 μmol/L BP <150/85 mmHg vs. < 180/105 mmHg	N = 31 with overt nephropathy (> 300 mg/l albuminuria) 8.4 yrs	Insufficient numbers to determine if BP control benefitted patients with overt nephropathy (P = 0.06)
Trocha et al. J Hypertension 1999 [12]	Type I DM Proteinuria > 0.5 g/d Cr < 265 μmol/L Diabetic retinopathy BP > 140/90 mmHg or need for BP meds BP self-monitoring with goal BP < 140/90 (IT) vs. routine care (RT)	N = 91 IT (N = 45) RT (N = 46) 10 yrs	Primary study endpoints (need for dialysis or death) decreased in IT group
Lewis et al. Am J Kidney Dis 1999 [14]	Type I DM with overt nephropathy Serum Cr < 354 μmol/L MAP of 92 mmHg (Group I) vs. 102–107 mmHg (Group II)	N = 129 N = 63 in Group I N = 66 in Group II 2 yrs	MAP 92 mmHg in Group I vs. 98 in Group II No significant difference in rate of decline of GFR Significant decrease in albuminuria in intensive group
Estacio et al. Diabetes Care 2000 [15]	Type II DM DBP > 90 mmHg CCr of 76 ml min-1 1.73 m^2^-1 Randomized to intensive therapy (IT) (DBP < 75 mmHg) or moderate therapy (MT) (DBP 80–89 mmHg)	N = 470 total N = 83 with overt proteinuria 5 yrs	BP decreased significantly with IT vs. MT (132/78 mmHg vs. 138/86 mmHg) Pts with overt albuminuria demonstrated a steady decline in creatinine clearance of 5–6 ml min-1 1.73 m^2^-1 yr-1 throughout the follow-up period in both IT and MT groups (pNS)

*categorical independent variables
